# Enhancing Lithium-Ion Battery Health Predictions by Hybrid-Grained Graph Modeling

**DOI:** 10.3390/s24134185

**Published:** 2024-06-27

**Authors:** Chuang Xing, Hangyu Liu, Zekun Zhang, Jun Wang, Jiyao Wang

**Affiliations:** 1 College of Electronics and Information Engineering, Sichuan University, Chengdu 610065, China; 2 School of Cyber Science and Engineering, Sichuan University, Chengdu 610207, China; 3 College of Biomedical Engineering, Sichuan University, Chengdu 610065, China; 4 Systems Hub, The Hong Kong University of Science and Technology (Guangzhou), Guangzhou 511453, China

**Keywords:** lithium-ion batteries, state-of-health, hybrid-grained temporal dependencies, deep learning

## Abstract

Predicting the health status of lithium-ion batteries is crucial for ensuring safety. The prediction process typically requires inputting multiple time series, which exhibit temporal dependencies. Existing methods for health status prediction fail to uncover both coarse-grained and fine-grained temporal dependencies between these series. Coarse-grained analysis often overlooks minor fluctuations in the data, while fine-grained analysis can be overly complex and prone to overfitting, negatively impacting the accuracy of battery health predictions. To address these issues, this study developed a Hybrid-grained Evolving Aware Graph (HEAG) model for enhanced prediction of lithium-ion battery health. In this approach, the Fine-grained Dependency Graph (FDG) helps us model the dependencies between different sequences at individual time points, and the Coarse-grained Dependency Graph (CDG) is used for capturing the patterns and magnitudes of changes across time series. The effectiveness of the proposed method was evaluated using two datasets. Experimental results demonstrate that our approach outperforms all baseline methods, and the efficacy of each component within the HEAG model is validated through the ablation study.

## 1. Introduction

Lithium-ion batteries, as a type of high energy density and lightweight energy storage device, are widely utilized in energy fields such as smartphones, laptops, drones, and electric vehicles [1,2]. However, the performance of lithium-ion batteries can deteriorate over repeated charge–discharge cycles, a phenomenon referred to as battery aging, resulting from physical and chemical changes caused by routine usage and operation [3]. Battery aging has the potential to lead to significant safety incidents and economic losses. State-of-health (SOH) refers to the ratio of a battery’s maximum capacity to its rated capacity, and it can also serve as a criterion for assessing battery aging [4,5]. SOH is primarily influenced by factors such as the number of charge–discharge cycles, charge–discharge rate, operating temperature, and internal resistance [6].

**Figure 1:**
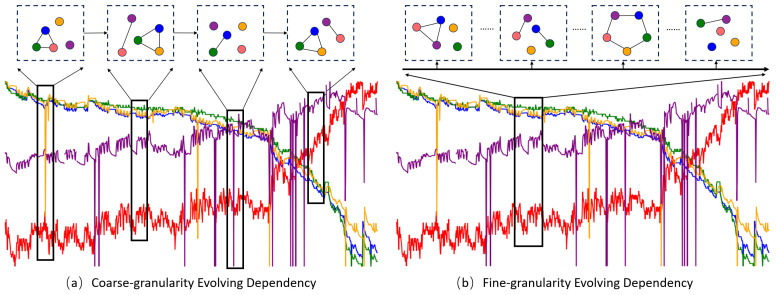
The possible dynamic interactions of variables in SOH prediction under two observation scales. The black box represents the sliding horizontal window, and the different colored spheres represent the representation vectors of different series learned by the model.

Current methodologies for predicting lithium-ion battery SOH are broadly classified into filter-based [7], machine learning [8], and deep learning techniques [9]. Filter techniques have limited capacity for handling large datasets, while machine learning requires intensive manual feature engineering and domain expertise, leading to potential labor intensiveness and suboptimal generalization. Deep learning traditionally struggles to capture dynamic temporal dependencies between input time series. As illustrated in Figure 1, dynamic dependencies typically manifest as either coarse-grained or fine-grained. Coarse-grained dependencies are akin to observing the overall trend over a long period, such as the daily temperature variation over a year, providing a broad view but missing finer details. Fine-grained dependencies focus on short-term variations, like hourly temperature changes within a day, capturing detailed fluctuations but potentially overlooking the broader trend. Dynamic graph networks are frequently employed in existing work to model evolving dependencies. However, predicting SOH is usually a long-term process influenced by intermittent factors [10]. Coarse-grained models struggle to capture sudden events and transient impacts in input time series, while fine-grained models find it challenging to capture the long-term dynamic temporal dependencies of SOH [11]. Consequently, both types of models may introduce certain errors in predicting SOH.

To address the limitations of existing models, we introduce the HEAG for predicting the lifespan of lithium-ion batteries. This model captures fine-grained evolving dependencies at each timestamp and integrates these insights into subsequent modeling stages. HEAG leverages a heterogeneous attention mechanism to aggregate fine-grained temporal information from multiple nodes on the graph. Additionally, our approach includes an FDG that relies on the Graph Attention (GAT) Mechanism. The FDG allows us to discern the interdependencies among various sequences at individual time nodes, capturing the patterns and magnitudes of variations within each sequence. Lastly, we deploy a Coarse-grained Dependency Graph generator to model temporal information across the entire timeline. This facilitates comprehensive modeling of the time series and enhances the understanding of mutual dependencies among different sequences.

In conclusion, the contributions of our work can be summarized as follows:

- In response to the limitations of traditional methods for predicting the health status of lithium batteries, we have introduced the HEAG model. This model utilizes hybrid-granularity time scales to significantly enhance predictive accuracy.
- To address the complex interplay of coarse-grained and fine-grained temporal dependencies within multiple input sequences more effectively, we have developed the FDG and the CDG. These tools help analyze the mutual dependencies across individual time points and entire time windows, offering new perspectives on heterogeneous correlation modeling among sequences.
- We have conducted extensive experiments using two publicly available datasets to demonstrate that the HEAG model can more accurately discern the correlations among various input sequences. This ability leads to improved predictions of the SOH of lithium batteries.

## 2. Related Works

### 2.1. Traditional Methods

Traditional methods for predicting the SOH of batteries are primarily classified into two categories: (1) filter-based techniques, and (2) machine learning approaches.

Filter-based models employ various techniques, including the Kalman filter [12,13], particle filter [14,15], and others. For instance, particle filters estimate SOH using stochastic models [16], while novel unbiased Kalman filters also assess battery SOH [17]. Additionally, extended Kalman filters with Lebesgue sampling [18] and adaptive methods considering parameter uncertainty using the Wiener process [19] have been proposed. Although these filter methods have significant advantages in self-correlation abilities, they have limitations when handling large datasets, especially in predicting the SOH of multiple battery cells [20].

In contrast, machine learning methods [21,22] are more effective at handling complex data and have been widely adopted for predicting battery SOH [23]. For example, ensemble learning [24], Gaussian process regression [25], and support vector regression [26] can all predict the SOH of lithium-ion batteries. Experimental results show that machine learning methods outperform filter-based approaches in terms of accuracy. However, machine learning also faces certain limitations. It is less capable of handling massive datasets compared to deep learning and relies on manually extracted features, complicating preprocessing and limiting the models’ generalizability in real-world applications [27].

### 2.2. Deep Learning-Based Methods

To effectively utilize the historical information from each battery unit, deep learning algorithms have been increasingly adopted [1,28]. These include CNN [29], RNN [30], LSTM [31], TCN [32], and Transformers [33].

CNN and LSTM combinations can identify battery aging features [34], and RNN models estimate the SOH of high energy density batteries [35]. You et al. designed an RNN-based degradation model for real-world driving data [36].

The BLS-LSTM hybrid model combines traditional statistical methods with neural networks, promoting battery health monitoring and energy management [37]. Optimized sliding windows and steady-state filtering reduce redundancy [38]. Multi-channel LSTM enhances flexibility in lifespan prediction, showcasing deep learning’s advantages in handling complex patterns and temporal dependencies [39]. Combining LSTM with Gaussian processes [40] or time series forecasting [41] leverages probabilistic and temporal modeling strengths. TCN addresses long-term dependencies, providing more accurate battery health predictions [42,43]. Integrating empirical mode decomposition with TCN reduces interference and enhances SOH prediction robustness [44]. Despite higher predictive accuracy than traditional methods, LSTM and TCN may overlook dynamic relationships between input sequences and SOH, leading to biases.

Transformers capture extensive dependencies and improve computational efficiency, advancing sequence modeling. Scholars like Chen [45] use denoising autoencoders to preprocess data for Transformer networks, while Luo [46] identified electrochemical impedance spectroscopy as a novel feature for Transformer-based SOH estimation. The Exponential Smoothing Transformer with Seasonal and Growth Embedding (SGEformer) has been proposed for predicting SOH [47]. These methods reflect the trend of integrating and advancing machine learning and deep learning for battery health assessment. However, Transformers, though adept at learning fine-grained inter-sequence relationships, face challenges in terms of high computational complexity and struggle to model coarse-grained relationships effectively.

### 2.3. Graph-Based Methods

While deep learning has demonstrated proficiency in handling complex data, it often struggles to unveil the inter-feature correlations essential for enhancing predictive model accuracy and robustness. Graph Convolutional Networks (GCNs) have gained prominence for their ability to analyze data relationships through undirected graphs, thus predicting SOH [48]. Derived models like MGCN [49] tackle complex network analysis in SOH prediction; GCN-DA [50] improves accuracy under multifactorial influences; CL-GraphSAGE [51] boosts scalability and efficiency for extensive data analysis; and CGGN-DCO [52] enhances efficiency in capturing complex temporal and spatial patterns. However, challenges such as MGCN’s high computational demand, GCN-DA’s cumbersome adaptation process, CL-GraphSAGE’s potential information loss, and CGGN-DCO’s complex training process may impede effective learning of health indicators tracking battery degradation. Our paper introduces the HEAG model, integrating coarse and fine-grained analyses for SOH prediction, achieving satisfactory results in computational complexity and predictive accuracy.

## 3. Methods

### 3.1. Problem Formulation

This study provides a tabulated summary and notation of key symbols in Table 1. We model the health state of lithium-ion batteries as a graph M=(Q,E), with Q embodying *N* factors influencing battery life, interconnected by E. Each factor *q* within Q has a dynamic input $x=\{x^{\left(1\right)},x^{\left(2\right)},\ldots ,x^{\left(t\right)}\}$, representing values over time. The variable *t* represents the cycle, which is the number of charge–discharge cycles of the battery. Each cycle impacts the SOH of the battery. Therefore, the SOH prediction problem is defined as a time series prediction problem because the SOH changes over time and with the number of cycles, with each cycle depending on the previous ones. Continuous cycles form a time series. After feature engineering, the refined input sequence is $X\in R^{L\times J}$, where *L* is the sequence length, and *J* is the number of dimensions at each time slot.

In our study, we delve into single-step prediction, which predicts the immediate future state using a historical data window. For input data *X* with a sliding window size *s*, we generate sub-sequences $X^{\left(t-s+1:t\right)}$ of length L−s and use the subsequent value $x^{\left(t+1\right)}$ as the target output *y*. Our goal is to craft a parameterized function F, which maps these sequences to future states, thereby enhancing prediction accuracy.

### 3.2. Model Architecture

**Figure 2:**
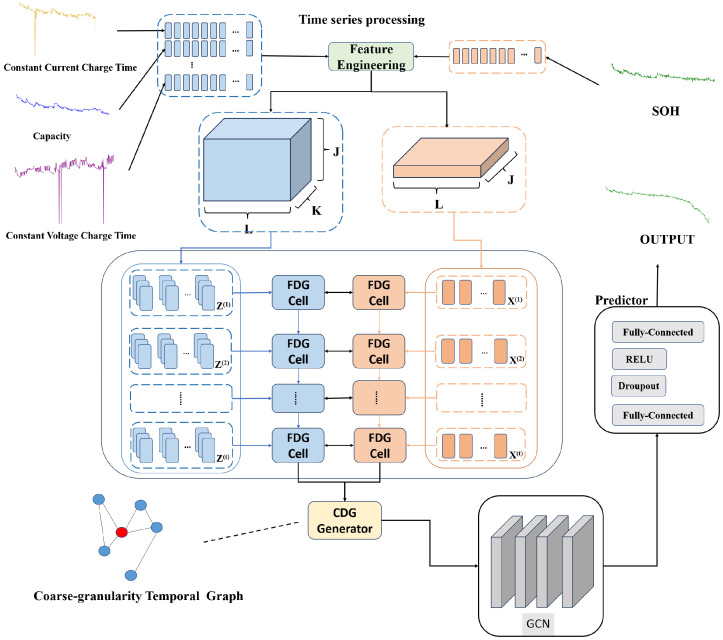
Structure and input/output dynamics of the hybrid network. Note: The time in the time series (such as Constant Current Charge Time, Capacity, Constant Voltage Charge Time, etc.) is represented by the cycle number.

The HEAG model’s structure, depicted in Figure 2, comprises four integral components: Graph and Time Series Processing, a Fine-grained Dependency Graph (FDG), a Coarse-grained Dependency Graph (CDG), and the Prediction module. The model employs time series processing to distill comprehensive features, setting the stage for advanced feature extraction. Through the intermediary steps involving the FDG and the CDG, the model culminates in the Prediction module, where it learns to project the feature vector $v^{\left(t+1\right)}$ onto the forecasted value ${\hat{y}}^{\left(t+1\right)}$.

#### 3.2.1. Feature Engineering

We derived two continuous features from each univariate sequence: (1) stability, represented by a normalized series, and (2) trend, depicted by a rate of change series. The computations for the normalized series and the rate of change series are detailed in Equation (1).

(1) $$\begin{array}{cc} x^{(i)\prime } & =\frac{x^{\left(i\right)}-\min\left(x^{\left(t-s+1:t\right)}\right)}{\max\left(x^{\left(t-s+1:t\right)}\right)-\min\left(x^{\left(t-s+1:t\right)}\right)},\\ x^{(i)\prime \prime } & =\left(x^{\left(i\right)}-x^{\left(i-1\right)}\right)/x^{\left(i-1\right)}. \end{array}$$

#### 3.2.2. Fine-Grained Evolving Aware Graph

In this section, we discuss efficient extraction methods for precise, fine-grained temporal dependencies using graph structures.

**Figure 3:**
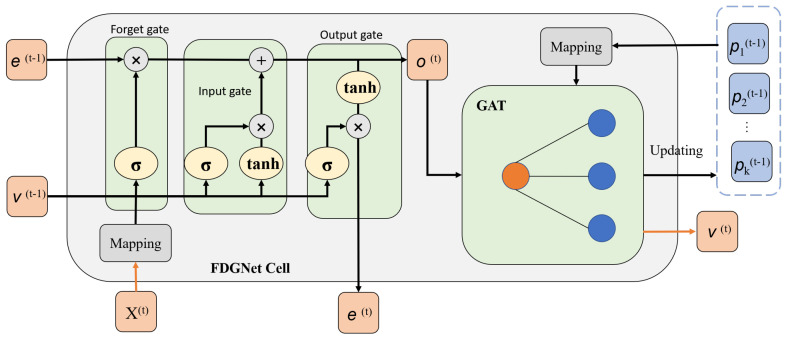
Cell structure of the FDG.

Given the flow sequence of the target influencing factors $X\in R^{L\times J}$ and the adjacent sequence $Z\in R^{K\times L\times J}$, K+1 independent FDG units are initialized for each sequence, with no parameter sharing between the FDG units. The architecture of the FDG unit is shown in Figure 3. Specifically, at timestamp *t*, $X^{\left(t\right)}$ and $Z^{\left(t\right)}$ are inputted into the first cell of their respective encoders. For instance, mapping $\left(x^{\left(t\right)},x^{(t)\prime },x^{(t)\prime \prime }\right)$ to a high-dimensional space generates a representation ${\mathrm{vector}}^{\left(t\right)}$ with size *J*. The state vector $e^{\left(1\right)}\in R^{1\times D}$ is randomly initialized at the first timestamp and continuously updated throughout the sequence. The temporally aware representation $v^{\left(t-1\right)}$ at the last timestamp is also considered as one of the inputs at timestamp *t*. By following the recurrent units based on the graph in Equation (2), the state vector is updated to $e^{\left(t\right)}$ at each timestamp and is prepared to enter the next cell. The temporal information of this input information flow at this timestamp is represented by the vector $o^{\left(t\right)}\in R^{1\times D}$.

(2) $$\begin{array}{cc} i^{\left(t\right)} & =\sigma (W_{i}^{T}\left[v^{\left(t-1\right)},{\hat{o}}^{\left(t\right)}\right]+b_{i}),\\ f^{\left(t\right)} & =\sigma (W_{f}^{T}\left[v^{\left(t-1\right)},{\hat{o}}^{\left(t\right)}\right]+b_{f}),\\ {\hat{e}}^{\left(t\right)} & =\tanh(W_{e}^{T}\left[v^{\left(t-1\right)},{\hat{o}}^{\left(t\right)}\right]+b_{e}),\\ g^{\left(t\right)} & =\sigma (W_{g}^{T}\left[v^{\left(t-1\right)},{\hat{o}}^{\left(t\right)}\right]+b_{g}),\\ e^{\left(t\right)} & =f^{\left(t\right)}⊙e^{\left(t-1\right)}+i^{\left(t\right)}⊙{\hat{e}}^{\left(t\right)},\\ o^{\left(t\right)} & =g^{\left(t\right)}⊙\tanh\left(e^{\left(t\right)}\right). \end{array}$$

Here, $i^{\left(t\right)}$ is the input gate, $f^{\left(t\right)}$ is the forget gate, and $g^{\left(t\right)}$ is the output gate. The learnable parameters at each gate, $W_{i}$, $W_{f}$, $W_{e}$, $W_{g}\in R^{J\times 2J}$, are accompanied by biases $b_{i}$, $b_{f}$, $b_{e}$, $b_{g}$. The symbol ⊙ indicates the element-wise product. After extracting temporal information at both neighbor and target encoder sides, the temporal representations of neighbor influencing factors $p^{\left(t\right)}\in R^{K\times D}$ and $o^{\left(t\right)}$ are inputted into the GAT module, which will be discussed in the next paragraph.

The Graph Attention (GAT) Mechanism was proposed to address the shortcomings of the Graph Convolutional Network (GCN), which struggles with inductive tasks on dynamic graphs and with capturing temporal dependencies due to its static topology. To capture fine-grained temporal dependencies and sustain influence from relevant influencing factors, the time attention score $\alpha_{i}^{\left(t\right)}$ between the target influencing factors and each relevant influencing factor is calculated and updated at each timestamp. Given the time representations of the target influencing factors and relevant influencing factors, the mechanism for information aggregation is depicted in Equation (3):

(3) $$\begin{array}{cc} h_{i}^{\left(t\right)} & =W_{q}^{T}g_{a}^{\left(t\right)}\times W_{k}^{T}p_{i}^{\left(t\right)},\\ \alpha_{i}^{\left(t\right)} & =\frac{h_{i}^{\left(t\right)}}{\sum_{i=1}^{K}h_{i}^{\left(t\right)}},\\ v^{\left(t\right)} & =ReLU(W_{fuse1}^{T}\left[o^{\left(t\right)},\sum_{i=1}^{K}\alpha_{i}^{\left(t\right)}W_{v}^{T}p_{i}^{\left(t\right)}\right]). \end{array}$$

In Equation (3), $W_{k}$ is the learnable weight of the key mapping function, $W_{q}$ is the weight of the query function, and $W_{v}$ is the weight of the value function. After a linear transform by $W_{k}$, the representation matrix of neighbor influencing factors is queried by the query vector, which is transformed from the representation of the target influencing factors. The attention scores facilitate the weighted aggregation of information from these neighbors. Following this, a fusion layer updates the temporal representation $v^{\left(t\right)}$ of the target influencing factors. Post-aggregation, the temporal information at timestamp *t* of the target influencing factors is also shared with neighboring factors, as detailed in Equation (4), where $W_{fuse2}$ denotes the weight of a new fusion layer.

(4) $$p_{i}\left(t\right)=RELU\left(W_{fuse2}^{T}\left[o\left(t\right),p_{i}\left(t\right)\right]\right)$$

The message passing operation is completed on each neighbor influencing factor to update their representations. Then, the updated temporal representation vectors of influencing factors at the present timestamp are inputted to the FDGNet cell for the next timestamp until the whole sequence is modeled.

#### 3.2.3. Coarse-Grained Dependency Graph

**Figure 4:**
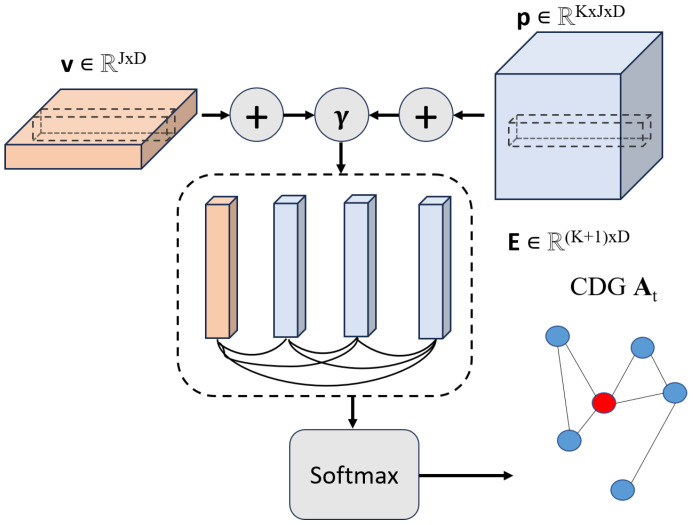
The structure of the CDG generator.

Acknowledging the inevitable loss of global information during the recurrent modeling process, the resulting fine-grained representation v proves inadequate. Previous studies have highlighted that fixed adjacency matrices might not accurately reflect the true connections between influencing factors. To address these issues, we have developed an implicit Coarse-grained Dependency Graph (CDG) generator that captures evolving global dependency information. Additionally, due to the continuous nature of the time series, simplistic pooling or naive addition of representations may either omit critical information captured in prior steps or introduce redundancy into the feature space. As illustrated in Figure 4, for representations at all time steps of both neighbor and target influencing factors p and v, we employ an addition operation with a computational attenuation coefficient γ, effectively aggregating information into space $E\in R^{(K+1)\times D}$. With v as an example, the process is formulated as follows:

(5) $$\begin{array}{cc} \gamma (t) & =\frac{1}{s-t},\\ E & =\mathrm{concat}(\left[\sum_{t\in s}\gamma^{\left(t\right)}v^{\left(t\right)},\sum_{t\in s}\gamma \left(t\right)p_{1}^{\left(t\right)},\ldots ,\sum_{t\in s}\gamma^{\left(t\right)}p_{N}^{\left(t\right)}\right]). \end{array}$$

Then, we apply a similarity matrix θ, where the similarity between each influencing factor $\theta_{ij}$ is measured by the inner product. Through a non-parameterized softmax function, a sparse adjacency matrix $A_{t}$ is computed as follows:

(6) $$A_{t}=\sigma ((\log\left(\theta_{ij}/\left(1-\theta_{ij}\right)\right).$$

Here, σ represents the activation function. Through the CDG generator, an auxiliary implicit graph is formed, capturing the global temporal representation. During each training iteration, $A_{t}$ is updated to maintain statistical alignment with the evolving dependency matrix, ensuring continual optimization post training. At the final time step *t*, the representations of $v^{\left(t\right)}$ and $p^{\left(t\right)}$, along with $A_{t}$, are fed into the graph convolutional network, updating the target influencing factors’ representation to $v^{{\left(t\right)}^{\prime }}$. This process facilitates achieving mixed granularity.

### 3.3. Result Prediction and Loss Function

The module utilizes a Multi-Layer Perceptron (MLP) for result prediction and loss analysis to generate predictions at time $\hat{y}$ at t+1. Specifically, the module consists of two linear neural layers, with a non-linear activation function and a dropout operation deployed between them. Through this MLP block, the output vector $v^{{\left(t\right)}^{\prime }}$ from the target influencing factor encoder is transformed into the prediction value at time ${\hat{y}}^{\left(t+1\right)}$ at t+1. Considering the robustness of HEAG in regression tasks, the smooth L1 loss is chosen as the loss function, defined as follows:

(7) $$L=\left\{\begin{array}{cc} 0.5\times {\left(y-\hat{y}\right)}^{2},\; & if\;|y-\hat{y}|\;<0.5\\ |y-\hat{y}|-0.5,\; & otherwise. \end{array}\right\}$$

## 4. Experiments

### 4.1. Battery Datasets

To enhance the credibility of the experimental results, we selected two publicly available datasets used in baseline methods for evaluation, ensuring that the samples were strictly chosen according to comparison criteria. These datasets were released by two different research institutions at different times. The dataset information statistics are shown in Table 2.

#### 4.1.1. NASA Dataset

**Figure 5:**
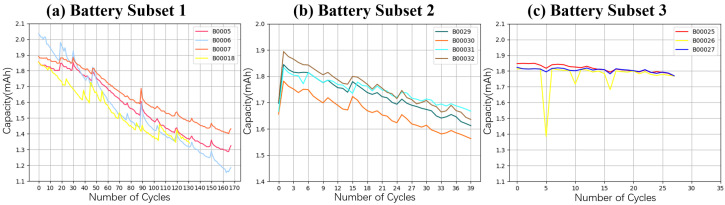
The capacity degradation trajectory of three battery subsets.

The HEAG model’s efficacy was validated using a battery dataset from NASA’s Ames Prognostics Center of Excellence [53], as cited in recent studies [50,54,55]. This dataset is divided into three subsets, with each subset encompassing specific batteries: Subset 1 features data from batteries numbered 5, 6, 7, and 18; Subset 2 from batteries 29, 30, 31, and 32; and Subset 3 from batteries 25, 26, and 27. These batteries were tested under three operational conditions: charging, discharging, and impedance, with detailed recordings of current, voltage, and temperature during operation. The charging protocol involved constant current (CC) charging at 1.5 A until the voltage hit 4.2 V, followed by constant voltage (CV) charging until the current fell to below 20 mA. The discharge settings varied slightly across experiments. The NASA dataset includes various features such as cycle number, ambient temperature, date/time, capacity, and the measurements of battery terminal voltage, battery output current, and battery temperature for each cycle, all of which are used as inputs for the model. The primary output from this model is the SOH. The repeated charging and discharging cycles cause accelerated degradation and capacity loss in lithium-ion batteries, as depicted in Figure 5, which illustrates the capacity degradation trajectories across the subsets.

#### 4.1.2. CALCE Dataset

The CALCE dataset is frequently utilized for research on SOH estimation [56,57]. It encompasses six subsets, known as CS2, which are differentiated by various cycle aging processes, such as CC–CV charging and CC discharge. In the charging stage, batteries charge under a constant current of 0.5 C until reaching a voltage of 4.2 V, and then they continue charging at this voltage until the current falls below 0.05 A. For discharging, the batteries discharge at various constant currents until the voltage falls to 2.7 V. This study focuses on four batteries within the dataset (CS2-35, CS2-36, CS2-37, CS2-38), all of which discharge at a constant rate of 1 C. These were examined using an Arbin battery tester. Within the CALCE dataset, the model inputs include date/time, cycle, capacity, resistance, CCCT (Constant Current Charging Time), and CVCT (Constant Voltage Charging Time), with the model providing predictions over a span of 72 cycles and providing output for 16 cycles.

### 4.2. Evaluation Metrics

To fairly assess the model performance, RMSE (Root Mean Square Error), MAE (Mean Absolute Error), and MedAE (Median Absolute Error) are utilized to measure the discrepancy between the actual values and the predicted values. Let $y_{n,l}$ denote the ground truth and ${\hat{y}}_{n,l}$ represent the predicted value. The definitions of these three metrics are as follows:

(8) $$MAE=\frac{1}{NL}\sum_{n=1}^{N}\sum_{l=1}^{L}\left|y_{n,l}-{\hat{y}}_{n,l}\right|,$$

(9) $$RMSE=\sqrt{\frac{1}{NL}\sum_{n=1}^{N}\sum_{l=1}^{L}{\left(y_{n,l}-{\hat{y}}_{n,l}\right)}^{2}},$$

(10) $$\mathrm{MedAE}=\mathrm{median}(|y_{n,l}-{\hat{y}}_{n,l}|).$$

### 4.3. Baseline Methods

To rigorously assess the efficacy of our novel model across diverse applications, we meticulously curated an assortment of both traditional and state-of-the-art benchmark methods for a comprehensive comparative study. These methodologies are broadly classified into sequence and graph-based models.

For sequence analysis, our arsenal included ARIMA [58] for forecasting time series data, linear regression (LR) [59] for tackling classification and regression challenges, GRU [60] for the intricate modeling and forecasting of sequential data, and the hybrid CNN+LSTM [34] model that fuses the strengths of CNNs and LSTMs for enhanced sequence data analysis. Furthermore, we explored the potential of TCN [43] and Transformer [61] models for advanced sequence-to-sequence learning, with the Transformer model employing a sophisticated self-attention mechanism for superior sequence data handling. The State-of-Health Prediction of Lithium-Ion Batteries using SGEformer [47] model was recently proposed, which integrates seasonal and growth embedding layers into the Transformer architecture, enhancing its ability to handle time-series data specifically tailored for battery life prediction.

Transitioning to graph-based models, we harnessed MGCN [49] to capture features at different scales by introducing multiple receptive fields and integrating these into a more robust feature representation, effectively enhancing feature representation and relationship mining among signals. Additionally, we referenced the GCN-DA [62] model, which incorporates a dual attention mechanism into a graph convolutional network to enhance the extraction and utilization of relevant features from time-series data. To further refine the quality of graph-influencing factor embeddings, we utilized CL-GraphSAGE [51], which leverages the strengths of both CNN and LSTM to extract temporal features, while the GraphSAGE component addresses spatial interdependencies. Additionally, the CGGN-DCO [52] model enhances the ability to capture temporal dependencies between features by integrating gated graph neural networks with dynamic convolutional operations.

### 4.4. Implementation Settings

During the implementation phase, the Adam optimizer was employed with a constant learning rate of 0.0001, and a batch size of 64 was established. The model underwent training over 20 epochs, with the hidden vector dimensionality steadfastly set at 64. To curb the risk of overfitting, a dropout rate of 0.1 was instituted. For the selection of the most effective parameter fusion, a grid search approach was harnessed, meticulously calibrating parameters within the validation dataset. This calibration included *K* values in the set [2,4,6,8], as well as *s* values of [11,16].

All experimental procedures were executed on a 64-bit Linux server, outfitted with an NVIDIA GeForce GTX 3090 GPU. We developed HEAG and the comparison baseline models utilizing Python 3.8 and PyTorch 1.7.0. Each experimental iteration was performed five times to ensure the statistical robustness of our findings.

For models lacking available official code in subsequent sections, we took the initiative to craft implementations independently; for those with accessible official code, we utilized it directly. To guarantee equitable comparisons, we meticulously tuned the parameters for all models, targeting their peak performance levels.

### 4.5. Comparison Results and Analysis

As shown in Table 3 and Table 4, in a detailed comparative analysis of SOH prediction models across the CALCE and NASA datasets, the HEAG distinguishes itself with exceptional performance. This model achieves the lowest RMSE and MAE, indicative of its advanced predictive abilities for battery health. Traditional methods like ARIMA lack the adaptability to capture intricate degradation patterns due to their linear nature and fall short in handling the nonlinearities present in battery usage data.

LR and GRU show improvements over traditional statistical methods but still face challenges with complex temporal sequences. These machine learning models often require expert domain knowledge for feature engineering, which is labor-intensive and which limits scalability.

Deep learning models, including CNN combined with LSTM, TCN, and Transformers, enhance the ability to understand sequential dependencies. They leverage the temporal information contained within the input data more effectively than traditional machine learning models. Despite this, their prediction accuracy, while better, is not as consistent or high-reaching as that of HEAG.

HEAG stands out not only against traditional deep learning approaches but also when compared to newer models such as SGEformer, MGCN, GCN-DA, CL-GraphSAGE, and CGGN-DCO. HEAG’s nuanced approach to temporal dependencies, utilizing both coarse- and fine-grained insights, enables a more sophisticated capture of SOH patterns in lithium-ion batteries.

HEAG’s robustness stems from its comprehensive approach to temporal analysis, blending the attention to detail seen in fine-grained analysis with the overarching patterns recognized by coarse-grained analysis. This dual approach allows the HEAG model to understand the immediate fluctuations and the long-term trends within the battery’s discharge cycles. This level of detail enables HEAG to capture both sudden, transient changes and long-term degradation patterns, enriching the model’s comprehension of battery health and its predictive precision.

In conclusion, while the deep learning models show significant potential, the hybrid approach taken by HEAG, with its ability to synthesize multi-scale temporal patterns, sets a new standard for accuracy and reliability in SOH prediction for lithium-ion batteries.

### 4.6. Ablation Study

For our comprehensive evaluation of the HEAG model, we placed special emphasis on the vital roles played by fine-grained and coarse-grained information in influencing the model’s performance metrics. To dissect these influences with greater precision, we conducted two key ablation experiments: one removing fine-grained information (labeled as HEAG w/o fine) and another eliminating coarse-grained information (labeled as HEAG w/o coarse). The purpose of these experiments was to delineate the individual and combined effects of these types of information on the model’s predictive accuracy.

**Figure 6:**
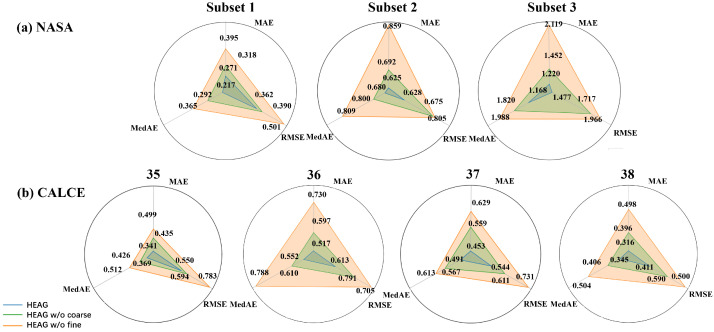
Ablation study of NASA and CALCE. The numerical unit is $10^{-2}$.

Insights drawn from Figure 6a,b, corresponding to the NASA and CALCE datasets, respectively, emphasize the significance of both types of information. Figure 6a clearly displays a performance reduction in key metrics such as RMSE, MAE, and MedAE by 17.5%, 23.5%, and 28.7%, respectively, on the NASA dataset following the removal of fine-grained and coarse-grained information. Similarly, Figure 6b, which focuses on the CALCE dataset, reveals decreases of 19.7%, 24.9%, and 20.1% in these metrics after the same omissions.

Our extensive analysis across these datasets not only confirms the negative impact of omitting any type of information on model performance but also highlights the model’s refined response to different dataset characteristics. This in-depth examination not only reaffirms the resilience and adaptability of the HEAG model but also provides deep insights into how fine-grained and coarse-grained information differentially and collectively influence model performance across various scenarios. Therefore, our study not only showcases the HEAG model’s strengths in complex predictive tasks but also establishes a detailed framework for future research to delve into the complex effects of model components on performance outcomes.

### 4.7. Case Study: Cross-Battery Evaluation

**Figure 7:**
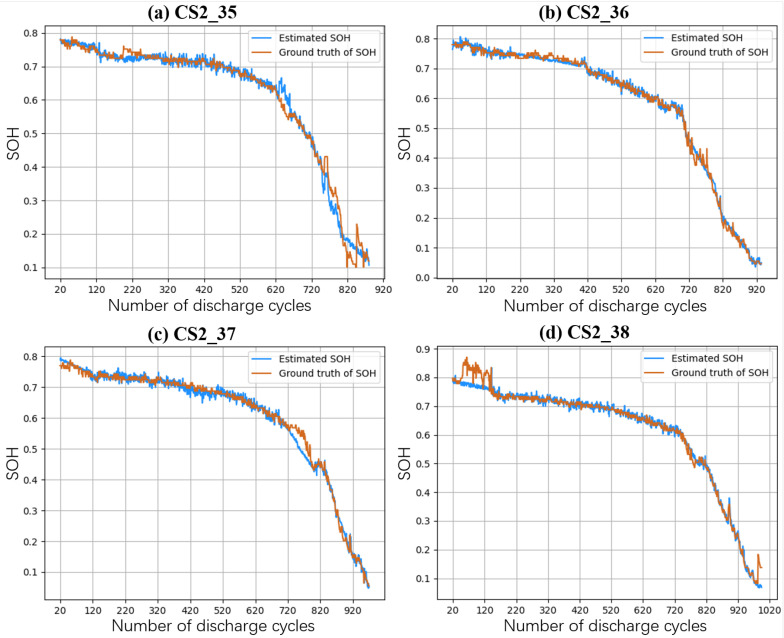
The SOH estimation results for batteries CS2-35, CS2-36, CS2-37, and CS2-38 within the CALCE dataset.

We utilized the CALCE dataset for cross-battery evaluation, involving four batteries. One battery was designated as the target test set, while the other three batteries were trained together. This process was repeated four times, with each battery serving as the target test set once. As shown in Figure 7a,b, the experimental results produced four graphs. By observing these graphs, we derived several observations.

Shape Analysis: The plots Figure 7a–d display the estimated SOH against the ground truth across the discharge cycles for four batteries. A consistent pattern is observed, with a plateau in the early cycles followed by a steeper decline, suggesting a characteristic battery degradation curve. However, some plots show divergence in this pattern, indicating potential improvements in capturing the lifecycle nuances of individual batteries.

Bias and Deviation: All graphs indicate some degree of bias, where the estimated SOH either overestimates or underestimates the ground truth. The deviation is more pronounced as the batteries near the end of their lifecycle, highlighting a challenge in accurately predicting the steepness and timing of SOH decline.

Anomaly Detection Ability: The predictive model’s ability to capture anomalous fluctuations is inconsistent. Initial fluctuations in SOH estimation, particularly in plot Figure 7d, reveal a sensitivity to anomalies, yet the model does not consistently detect all abrupt changes, suggesting a need for refined modeling techniques to improve prediction accuracy and reliability.

Overall, while the model shows promise in generalizing across different batteries, the case study reveals limitations in shape adherence, bias accuracy, and anomaly detection, guiding future model enhancements.

## 5. Conclusions

In this paper, we present a novel evolving aware graph model designed to predict the health status of lithium-ion batteries. This model, known as HEAG, integrates both Fine-grained Dependency Graph (FDG) and Coarse-grained Dependency Graph (CDG) components to discern temporal dependencies at various granularities. Specifically, the FDG captures dependencies between sequences at individual time points, while the CDG perceives dependencies across entire time series. Extensive experiments were conducted using publicly available datasets containing discharge cycle data such as current, voltage, temperature, and other parameters. Prior to experimentation, feature engineering was employed to optimize efficiency and accelerate convergence. The results demonstrate that our method accurately identifies dependencies between different time series and predicts the SOH with high precision. Compared to the baseline methods and four new methods discussed in this paper, our approach shows superior performance in terms of RMSE, MAE, and MedAE. Additionally, the necessity of each component was validated through ablation studies, and cross-evaluation case studies of the batteries yielded favorable outcomes. Moving forward, the effectiveness of the proposed method will be assessed using various types of lithium-ion battery datasets to confirm its broader applicability.

## Figures and Tables

**Table 1:** A summary of symbols and descriptions.

Symbol	Description
M	The SOH of lithium-ion batteries.
Q	Factors related to the state-of-health of lithium-ion batteries.
E	The interplay of influences among various factors.
*N*	The number of influencing factors on battery life.
*L*	The length of the information flow.
*J*	The number of subseries after feature engineering.
*X*	The finalized input series of each computational influencing factor.
*D*	The dimension of the hidden vector.
E	The space after information aggregation.
*Z*	The input series of graph-based neighbor influencing factors.
*K*	The number of input sequences.
*s*	The size of the sliding historical window.
$A_{t}$	The global temporal sparse adjacency matrix.
W	The learnable parameter of the neural layer.
b	The bias of the neural layer.
*x*	The original series of the adjacent influencing factors.
$x^{\prime }$	The normalized series of the adjacent influencing factors.
$x^{\prime \prime }$	The trend series of the adjacent influencing factors.
*X*	The integrated input series of the adjacent influencing factors.
*y*	The ground truth of each training series.
$\hat{y}$	The output of the HEAG.
e	The state vector in the HEAG.
o	The output temporal representation vector.
p	The representation vectors of adjacent influencing factors.
v	The final temporal representation vector for prediction.
γ	The attenuation coefficient.
α	The attention weight learned by the attention mechanism.
L	The loss function used in this work.

**Table 2:** Dataset information statistics.

Dataset	Battery Pack	Sequence Number	Average Length
CALCE	CS2-35	25	6735
	CS2-36	26	5639
	CS2-37	27	7968
	CS2-38	28	8236
NASA	5-6-7-18	4	857
	25-26-27-28	4	739
	25-44	20	821
	45-46-47-48	4	769
	49-50-51-52	4	807
	53-54-55-56	4	792

**Table 3:** Experimental data based on the CALCE dataset. The bold font highlights the superior performance of the HEAG model compared to other methods.

Method	Metrics	35	36	37	38	Average
ARIMA [58]	RMSE ($10^{-2}$)	51.060	53.177	49.738	42.691	49.166
	MAE ($10^{-2}$)	48.405	50.608	48.971	39.931	46.979
	MedAE ($10^{-2}$)	49.334	52.361	50.059	41.798	48.388
LR [59]	RMSE ($10^{-2}$)	49.200	46.736	46.493	41.890	46.080
	MAE ($10^{-2}$)	45.483	46.905	46.984	38.005	44.344
	MedAE ($10^{-2}$)	42.103	47.082	43.233	39.777	43.049
GRU [60]	RMSE ($10^{-2}$)	32.536	34.613	31.611	25.665	31.106
	MAE ($10^{-2}$)	29.296	31.215	28.718	23.943	28.293
	MedAE ($10^{-2}$)	30.440	34.504	32.136	25.727	30.702
CNN+LSTM [34]	RMSE ($10^{-2}$)	27.635	30.620	28.127	23.033	27.354
	MAE ($10^{-2}$)	26.231	30.240	27.436	19.939	25.961
	MedAE ($10^{-2}$)	27.303	31.420	29.454	21.129	27.326
TCN [43]	RMSE ($10^{-2}$)	12.298	12.909	11.937	9.272	11.604
	MAE ($10^{-2}$)	10.673	12.290	10.371	8.063	10.349
	MedAE ($10^{-2}$)	10.970	13.183	10.409	8.923	10.871
Transformer [61]	RMSE ($10^{-2}$)	16.033	19.065	17.914	13.219	16.558
	MAE ($10^{-2}$)	14.899	16.386	15.132	12.901	14.830
	MedAE ($10^{-2}$)	15.017	19.260	16.433	13.123	15.958
SGEformer [47]	RMSE ($10^{-2}$)	1.992	2.730	2.083	1.747	2.138
	MAE ($10^{-2}$)	1.242	2.153	1.583	0.916	1.473
	MedAE ($10^{-2}$)	1.866	2.397	1.999	1.158	1.855
MGCN [49]	RMSE ($10^{-2}$)	4.157	6.689	4.689	3.443	4.745
	MAE ($10^{-2}$)	2.866	5.343	3.321	2.210	3.435
	MedAE ($10^{-2}$)	3.005	6.096	4.343	2.948	4.098
GCN-DA [50]	RMSE ($10^{-2}$)	1.849	4.885	1.942	1.808	2.621
	MAE ($10^{-2}$)	1.021	3.096	1.696	0.915	1.682
	MedAE ($10^{-2}$)	1.721	3.784	2.014	1.540	2.265
CL-GraphSAGE [51]	RMSE ($10^{-2}$)	0.987	1.127	1.015	0.893	1.005
	MAE ($10^{-2}$)	0.755	1.082	0.863	0.570	0.817
	MedAE ($10^{-2}$)	0.720	0.952	0.719	0.598	0.747
CGGN-DCO [52]	RMSE ($10^{-2}$)	0.783	0.913	0.810	0.623	0.782
	MAE ($10^{-2}$)	0.544	0.755	0.530	0.339	0.542
	MedAE ($10^{-2}$)	0.659	0.820	0.634	0.407	0.630
HEAG	RMSE ($10^{-2}$)	**0.550**	**0.613**	**0.544**	**0.411**	**0.530**
	MAE ($10^{-2}$)	**0.341**	**0.517**	**0.453**	**0.316**	**0.407**
	MedAE ($10^{-2}$)	**0.369**	**0.552**	**0.491**	**0.345**	**0.439**

**Table 4:** Experimental data based on the NASA dataset. The bold font highlights the superior performance of the HEAG model compared to other methods.

Method	Metrics		Subset 1				Subset 2				Subset 3		AVG
		No. 5	No. 6	No. 7	No. 18	No. 29	No. 30	No. 31	No. 32	No. 25	No. 26	No. 27	
ARIMA	RMSE ($10^{-2}$)	14.207	38.154	37.087	21.044	19.410	10.231	9.067	11.635	16.454	20.119	38.599	21.455
	MAE ($10^{-2}$)	13.844	37.469	36.899	19.098	19.451	8.060	8.716	13.000	16.365	17.497	38.552	20.814
	MedAE ($10^{-2}$)	14.844	38.369	37.850	18.218	19.275	8.528	8.382	13.306	16.203	15.470	38.616	20.824
LR	RMSE ($10^{-2}$)	13.170	36.336	36.179	19.635	19.022	8.131	8.623	11.882	15.611	19.906	38.189	20.608
	MAE ($10^{-2}$)	12.404	36.878	36.147	18.005	17.593	8.024	6.746	11.063	15.813	16.665	37.195	19.685
	MedAE ($10^{-2}$)	12.973	37.465	38.234	16.688	19.260	6.884	8.004	12.460	14.151	13.432	36.446	19.636
GRU	RMSE ($10^{-2}$)	8.461	31.791	32.679	15.876	15.538	4.979	3.463	8.341	11.529	16.423	34.194	16.661
	MAE ($10^{-2}$)	7.365	32.962	32.312	13.307	15.298	3.669	3.103	7.923	10.544	12.116	32.873	15.588
	MedAE ($10^{-2}$)	9.894	33.889	34.181	14.124	15.502	4.476	4.082	8.526	10.080	10.410	33.348	16.229
CNN + LSTM	RMSE ($10^{-2}$)	7.249	30.620	30.550	13.280	12.620	2.371	2.181	5.478	9.216	13.700	31.490	14.432
	MAE ($10^{-2}$)	6.047	30.240	30.980	11.850	12.450	1.939	1.556	5.324	8.975	10.020	31.440	13.711
	MedAE ($10^{-2}$)	7.420	31.420	31.060	11.170	12.770	1.634	1.266	5.663	8.899	7.505	31.230	13.640
TCN	RMSE ($10^{-2}$)	2.174	2.468	2.445	2.440	2.321	2.722	2.406	3.074	3.639	3.737	3.064	2.772
	MAE ($10^{-2}$)	1.886	2.291	2.434	2.521	2.375	2.584	2.375	3.148	3.466	3.123	3.050	2.660
	MedAE ($10^{-2}$)	1.980	2.197	2.449	2.262	2.306	2.656	2.423	3.193	3.415	3.039	3.127	2.641
Transformer	RMSE ($10^{-2}$)	4.520	25.030	25.344	4.856	7.857	4.049	2.069	1.931	2.618	23.050	8.035	9.942
	MAE ($10^{-2}$)	3.618	16.610	16.325	3.877	4.603	3.491	1.826	1.590	2.244	18.240	7.348	7.252
	MedAE ($10^{-2}$)	2.751	13.260	13.449	3.224	2.064	3.176	1.723	1.272	2.202	11.580	6.390	5.554
SGEformer	RMSE ($10^{-2}$)	0.987	1.547	1.483	1.421	1.258	1.946	1.415	2.366	2.629	2.680	2.021	1.796
	MAE ($10^{-2}$)	1.225	1.532	1.559	1.279	1.211	1.798	1.647	2.392	2.485	2.475	1.844	1.768
	MedAE ($10^{-2}$)	1.267	1.097	1.196	1.360	1.512	2.053	1.258	2.438	2.625	1.954	1.940	1.700
MGCN	RMSE ($10^{-2}$)	0.659	1.689	1.877	1.268	0.491	1.159	0.472	1.049	1.675	2.292	1.645	1.298
	MAE ($10^{-2}$)	0.538	1.343	1.460	1.018	0.416	1.118	0.416	1.034	1.552	1.544	1.627	1.097
	MedAE ($10^{-2}$)	0.478	1.096	1.167	0.934	0.384	1.139	0.418	1.012	1.517	1.080	1.639	0.988
GCN-DA	RMSE ($10^{-2}$)	0.849	1.129	1.012	1.518	1.351	1.866	1.188	1.849	2.414	2.533	1.909	1.602
	MAE ($10^{-2}$)	1.004	1.038	1.010	1.211	0.942	1.625	1.104	2.091	2.401	1.867	1.940	1.476
	MedAE ($10^{-2}$)	0.774	0.975	1.137	1.160	1.278	1.457	1.098	2.005	2.372	1.797	1.753	1.437
CL-GraphSAGE	RMSE ($10^{-2}$)	0.830	1.116	1.267	1.299	1.092	1.596	1.358	1.826	2.347	2.672	1.770	1.561
	MAE ($10^{-2}$)	0.723	1.079	1.181	1.338	1.157	1.457	1.162	2.038	2.131	1.887	1.726	1.443
	MedAE ($10^{-2}$)	0.704	0.971	1.271	1.151	1.103	1.524	1.116	1.993	2.196	1.754	1.945	1.430
CGGN-DCO	RMSE ($10^{-2}$)	0.228	0.513	0.501	0.711	0.480	0.980	0.567	1.274	1.686	1.941	1.157	0.913
	MAE ($10^{-2}$)	0.164	0.355	0.460	0.553	0.406	0.864	0.489	1.243	1.566	1.346	1.129	0.780
	MedAE ($10^{-2}$)	0.120	0.220	0.475	0.477	0.433	0.881	0.466	1.259	1.542	1.146	1.166	0.744
**HEAG**	**RMSE ($10^{-2}$)**	**0.122**	**0.357**	**0.382**	**0.587**	**0.332**	**0.848**	**0.423**	**1.117**	**1.575**	**1.831**	**1.024**	**0.782**
	**MAE ($10^{-2}$)**	**0.109**	**0.235**	**0.311**	**0.431**	**0.257**	**0.755**	**0.356**	**1.133**	**1.418**	**1.244**	**0.997**	**0.659**
	**MedAE ($10^{-2}$)**	**0.118**	**0.101**	**0.322**	**0.325**	**0.318**	**0.762**	**0.309**	**1.125**	**1.430**	**1.043**	**1.031**	**0.626**

## Data Availability

The NASA and CALCE datasets used in this study can be accessed at https://www.nasa.gov/content/prognostics-center-of-excellence-data-set-repository and https://calce.umd.edu/battery-data, respectively, with access dates of 16 January 2024.

## Abbreviations

The following abbreviations are used in this manuscript:

SOH	State-of-health
FDG	Fine-grained Dependency Graph
CDG	Coarse-grained Dependency Graph
HEAG	Hybrid-grained Evolving Aware Graph
CNN	Convolutional Neural Network
RNN	Recurrent Neural Network
GRU	Gated Recurrent Network
LSTM	Long Short-term Memory
GAT	Graph Attention
TCN	Temporal Convolutional Network
CC	Constant Current
CV	Constant Voltage
MLP	Multi-Layer Perceptron
